# Thermal Deformations of Thermoplast during 3D Printing: Warping in the Case of ABS

**DOI:** 10.3390/ma14227070

**Published:** 2021-11-21

**Authors:** Jakub Ramian, Jan Ramian, Daniel Dziob

**Affiliations:** 1 Faculty of Mechanical Engineering and Robotics, AGH University of Science and Technology, 30-059 Krakow, Poland; ramian@student.agh.edu.pl; 2 Faculty of Medical Sciences in Katowice, Medical University of Silesia, 40-055 Katowice, Poland; ramianjann@gmail.com; 3 Department of Pharmaceutical Biophysics, Faculty of Pharmacy, Jagiellonian University Medical College, 30-688 Krakow, Poland

**Keywords:** 3D printing, warping, deformation, ABS

## Abstract

This research focuses on thermal deformations of thermoplast during three-dimensional printing. A filament acrylonitrile butadiene styrene was used, and the main focus was put on warping. Twenty-seven cuboids divided in six categories by their length, height, surface area, color, nozzle temperature and bed temperature were printed by Fused Filament Fabrication 3D printer. The whole process was captured by a thermal camera and the movies were used to analyze the temperature distribution during printing. All printouts were measured and scanned with a 3D scanner in order to highlight any abbreviations from the original digital models. The obtained results were used to formulate some general conclusions on the influence of selected parameters on the warping process. Based on the outcomes of the study, a set of guidelines on how to minimalize warping was proposed.

## 1. Introduction

In recent years, polymers, known to most as plastics, have become infamous for their environmental impact. In spite of this, polymers have gained an important spot in one branch of engineering, 3D printing. This is a relatively new and fast-developing manufacturing technology. Although the history of 3D printing started in the 1980s [1], in recent years it has experienced rapid growth in market share, from USD 11.56 billion in 2019 to USD 13.78 billion in 2020 [2], and is becoming more and more popular every day. There is good reason for that, since this technology offers many advantages, making it ideal for certain tasks, the most glaring one being that three-dimensional printing is uniquely suited to the production of small numbers of geometrically complicated parts. This made it synonymous with rapid prototyping. No other technology offers going from a computer model to real-life object so quickly and easily. In contrast to traditional methods, 3D printing is an additive manufacturing, which means that final products are made by adding layer after layer of material, rather than removing it, such as in CNC machining. Rapid prototyping was quickly adopted by the aerodynamics, automotive, and medicine sectors [3] due to the fact that it allows a large degree of design freedom and fast printing of cheap prototypes to easily implement any necessary changes. This makes it perfect for customization on a personal level, so that the product matches the recipient—whether it is a patient in need of hearing aids or an engine. This study focuses on the fused filament fabrication (FFF) type of 3D printing, commonly known as fused deposition modeling (FDM), that uses thermopolymers.

Thermopolymers are being used in a form of wire, commonly known as filament (Latin: filamentum—long appendix). Three most common filaments in 3D printing are ABS (acrylonitrile butadiene styrene), PLA (polylactide), and PETG (poly(ethylene terephthalate). Acrylonitrile butadiene styrene is an amorphous thermoplastic with a melting temperature low enough (210–270 °C) to be 3D printed. It consists of three monomers: acrylonitrile, butadiene and styrene. ABS is renowned for its heat and impact resistance, while being susceptible to alcohols, allowing easy post-production processing. Due to its properties it is widely used in many applications, including automotive, tools and toys. One significant drawback of this material is that ABS is strongly affected by thermal shrinking. PLA is an environmentally friendly and more flexible alternative but with poor heat resistance. PETG, on the other hand, is excellent for physical manipulation, such as drilling and sanding, while being impervious to chemicals and weaken by heat [4,5,6].

**Figure 1:**
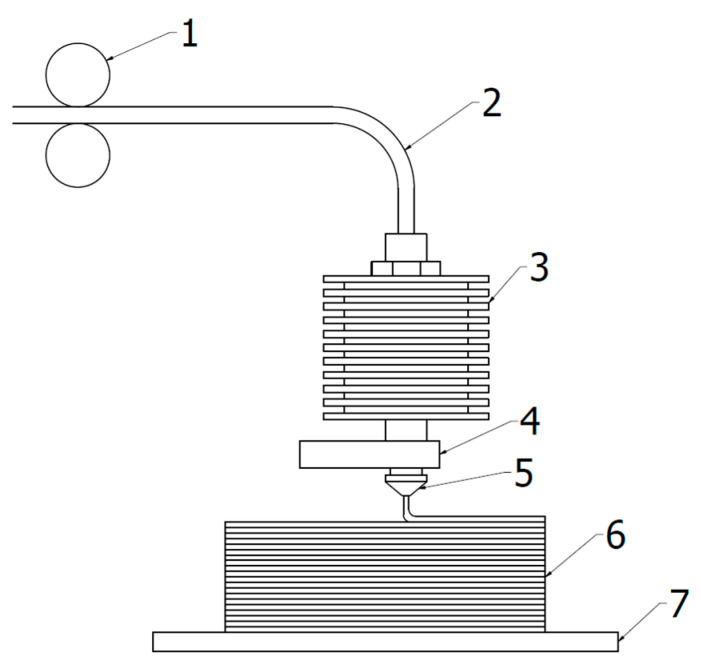
Schematic drawing of 3D printing projection. 1—extruder, 2—filament, 3—heatsink, 4—heatblock, 5—nozzle, 6—printing object, 7—heated bed.

In 3D fused deposition modeling printing, the filament is pulled into the heated nozzle, where it melts (see Figure 1). The nozzle moves, arranging filament on the bed layer by layer, first on a heated bed (heat improves adhesion properties of the plastic), then on top of previous layers, which are then fused together during the crystallization process, forming a given shape. G-code files uploaded to the printer are sets of simple instructions. They contain information on the temperatures of the nozzle and the heated bed, extruder speed, coordinates for the nozzle movement, and other necessary parameters. These files are created using slicer computer programs. They slice 3D models into sets of 2d planes along the *x* and *y* axes, which are calculated along the necessary filament feeding instructions of the site, that is, the program calculates how much filament should be given to the printer at a given moment to create the desired shape [7]. Due to accessibility, low cost, and simplicity FDM becomes more and more popular-nowadays it is utilized not only by small companies and scientists but also by enthusiasts for personal usage.

However, FDM 3D printing has limitations. Some of them result from the nature of the materials used, e.g., thermoplastics, which due to limitations of most common desktop 3d printers must be formed at around 180–270 °C. This is echoed in the properties of prints. Excluding ABS, prints made of common materials (those under 270 °C, possible to make on cheap printers) rapidly degrade mechanical performance as temperature rises, even at relatively low temperatures. Another characteristic of FDM printing is a result of its layered structure: prints are built from lines as wide as the diameter of a nozzle (usually 0.4 mm) and as high as 0.1 to 0.2 mm. This, together with temperature-related size changes, means that the difference between the CAD model and the final printout can usually be measured in tenths of millimeters [8]. Another problem is the limited area in which the whole object is printed [9]. The often-overlooked problem with rapid development is the fact that the whole process is full of variables (nozzle and bed temperature, infill level, kind of filament, retraction rate, nozzle movement) that without a proper and meticulous maintenance process will fail. It is interesting that, due to easy accessibility to printers, the betterment of 3D printing is not the domain of scientists—hundreds of movies about 3D printing from self-made experts can be found on the Internet. However, this growing popularity has also reflected in an increasing number of scientific studies. Some of these issues were investigated more rigorously than others. Mendes [10] tackled the problem of filament flow, Schirmeister [11] developed methods to improve bed adhesion, Trejo [12] focused on layer-to-layer adhesion, Wu [13] shortened printing time, Soares [14] improved final quality of products. However, even when all parameters are properly calibrated and controlled, some unexpected problems may still occur. Most notably are changes in the dimensions of the model, a process called warping.

**Figure 2:**
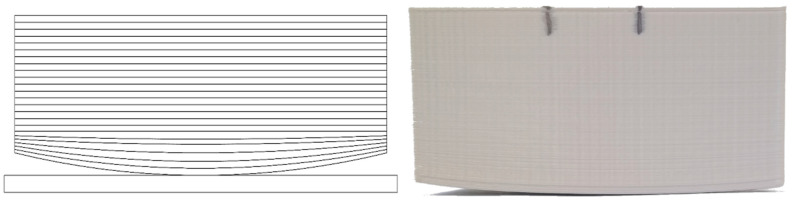
Warping. (**Left**): schematic drawing shows all layers. (**Right**): photo of a real printout.

**Figure 3:**
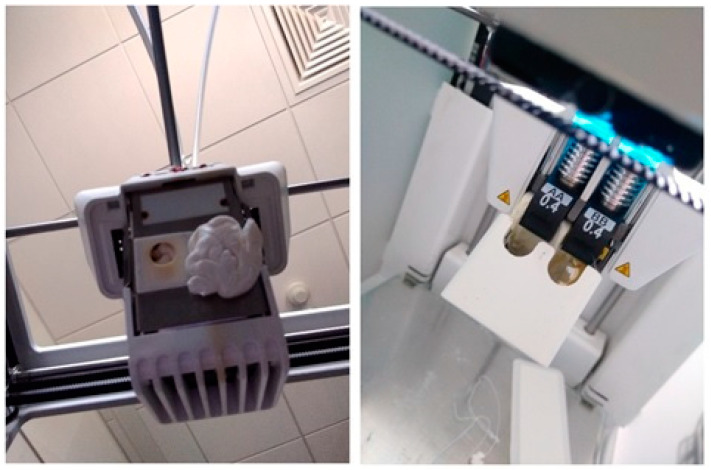
Printer’s nozzle damaged by a filament which did not adhere due to warping.

Warping is a deformation caused by temperature changes between neighboring layers of the printout, as they cool and harden during printing, or between the first layer and the printing stage (bed), resulting in material shrinking and contracting, most notably around the bottom corners. Schematically, it is shown in Figure 2. In extreme cases, warping may even damage the printer, when filament adherence to printer’s bed is weakening by warping to such degree that the filament peels off the bed and sticks to the nozzle, sometimes blocking it completely (Figure 3).

Problems may occur according to two mechanisms: when the filament cools to a glass transition temperature (i.e., the temperature at which half of the change in heat capacity occurs [15]) while exiting the nozzle and during the transition from glass transition temperature to chamber temperature [7]. This phenomenon is nothing new in the world of 3D printing, and in past years many researchers focused on this problem.

There are several methods of combating warping. The use of epoxy resin-based adhesive layers on the printing stage performed by Nazan reduces warping by 10% [16]. Other work by the same author pointed out that the optimal thickness of the layer is 0.3 mm [17]. Basavaraj proposed a method of printing at a 30° angle between the long axis of the nozzle and the bed [18], however, this requires additional scaffolding. The mathematical description of warping can be found in other papers [7].

The aim of this paper was to present practical, basic, systematic study on how basic parameters of 3D printing and physical variabilities of shape of the printed objects influence the thermal deformations of the ABS printouts.

## 2. Materials and Methods

As a thermoplastic polymer, acrylonitrile butadiene styrene (ABS), which is one of the most popular materials, was used for this study. It consists of three types of monomers in variable proportions: 50–60% of styrene, 10–25% of butadine, and 20–30% of acrylonithrile. It may also contain additions such as pigments and other substances to improve print quality, surface finish or lower the printing temperatures. The main advantage of ABS in 3D printing is its relatively high glass transition temperature (~105 °C) that allows the printouts to stay dimensionally stabile in the temperatures high enough to deform prints made of other popular materials. Another useful feature is that ABS is hard and resistant to scratches, impact-resistant but susceptible to aromatic hydrocarbons, halogenated hydrocarbons and alcohols, which allows for easy processing. Nowadays it is one of the most popular filaments, despite the fact that it is susceptible to warping.

The ABS filament used throughout the study was made by Fiberlogy (Brzezie, Poland). It contains ≤98% of acrylonitrile butadiene styrene (CAS. 9003-56-9) and less than 2% of additions [19]. The characteristics specified by the manufacturer are presented in Table 1 [19].

As was mentioned in the introduction, warping is caused by uneven temperature distribution of the object during its cooling (which also occurs while the model is still being printed). Uneven distribution of heat causes uneven shrinkage. Problem of thermal shrinkage is present in many other manufacturing techniques, but it is specially affecting the 3D printing due to its additive nature. Adding a layer after layer pf semi-molten material causes delays in solidification of material in different parts of the object. This can vary depending on geometry, specific material and printing setting. Therefore, the general idea of our research was to print various cuboids and register their temperatures with a thermal imaging camera. That allows us to observe temperature changes on the surface of printed cubes, which gives the ability to show temperature changes during whole process of warping.

**Figure 4:**
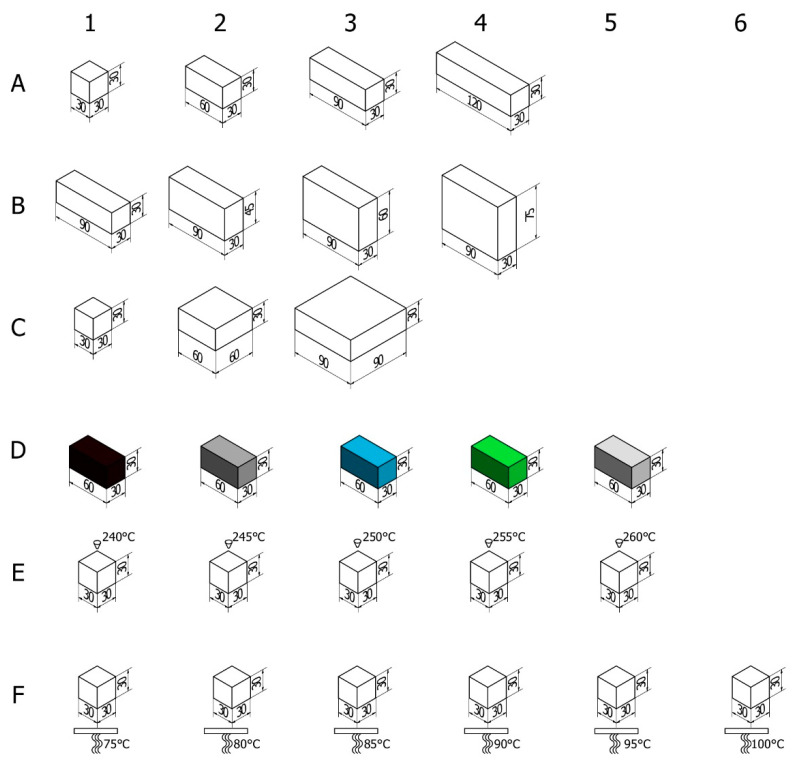
Graphics of various printouts. Group (**A**) changes length, group (**B**) changes height, group (**C**) surface area, group (**D**) color, (**E**) nozzle temperature, and (**F**) bed temperature.

We have prepared and printed various cuboids in order to verify how respective parameters influence the final degree and type of deformation. We have started with the cube 30 × 30 × 30 mm^3^, nozzle temperature 255 °C and bed 90 °C and change, respectively (see Figure 4 and Table 2):

(a) Length,
(b) Height,
(c) Surface area,
(d) Color of the ABS, to determine if pigments can significantly affect the warping,
(e) Temperature of the nozzle and
(f) Temperature of the printing stage.

All object files were prepared in the Autodesk Inventor 2021 environment (Autodesk, Inc., San Rafael, CA, USA) and then sliced using Ultimaker Cura 4.11 (Ultimaker, Utrecht, The Netherlands). At the pre-study stage we carried out a test of uniformity. In this test series five cuboids with diameters 30 × 30 × 30 mm^3^ and 30 × 30 × 60 mm^3^ were printed one after the other in the same external conditions, in order to determine the impact of warping and the spread of the results between trials. For both sizes the spread of mean warping results between samples is much smaller than the uncertainty associated with different heights of the edges within the same sample, so in the reminder of the study only one example per print was done, since our priority was to print the whole series from the same spool of filament.

**Figure 5:**
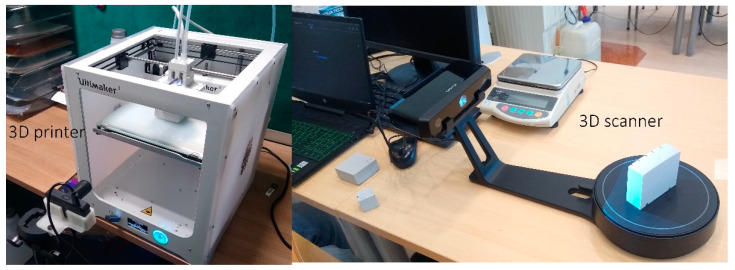
Experimental setup. (**Left**): 3D printer with infrared camera placed before. (**Right**): 3D scanner with the object on it.

All models were printed with an Ultimaker 3 printer with a 0.4 mm nozzle, moving at a speed of 60 mm/s with 20% infill, in the glass stage covered with an adhesive sheet to ensure consistent conditions [20]. As visible on Figure 5 Ultimaker 3 printer used in this study did not feature heated or closed chamber, as too better reflect majority of budget printers, used by many researchers. This is in contrast to a series of studies on warping which concentrate on much more advanced, and therefore more expensive printers [12,13,16,18,21,22,23]. With a cheaper device, colder air is circulating around the printout, increasing warping. Temperature distribution was registered throughout the printing process with the Flir One Pro LT camera (Teledyne FLIR LLC, Wilsonville, OR, USA) [24]. The geometric dimensions of the printed cuboids were measured with EinScan-SP SPECS Desktop 3D Scanner (Shining 3D, Hangzhou, China) [25] and then compared with those set in the project. The experimental setup is presented in Figure 5. All cubes were marked on the edges to allow easier assembly in the scanner software.

## 3. Results

### 3.1. Eye Inspection

**Figure 6:**
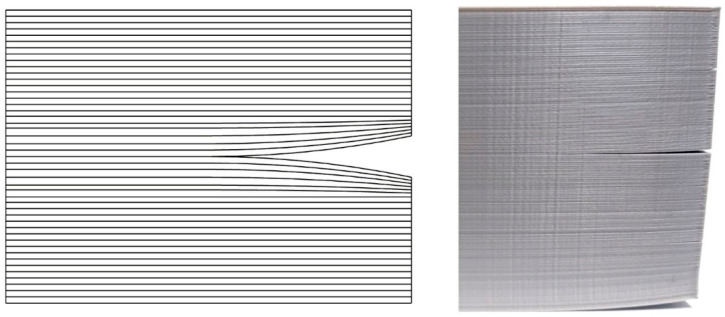
Crack stratifications of filament layers. (**Left**): schema; (**Right**): photo of real printout.

**Figure 7:**
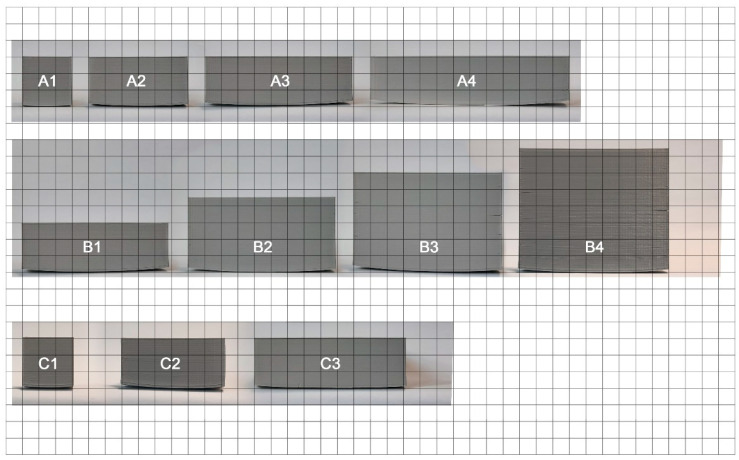
Photos of printed cubes for group A to C, placed on a 1 × 1 cm^2^ grid.

**Figure 8:**
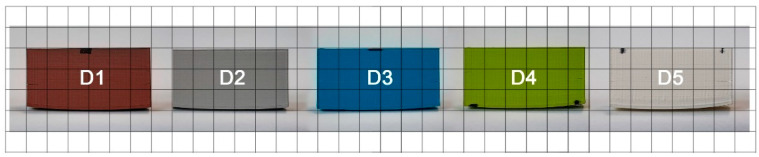
Photos of printed cubes for group D, placed on a 1 × 1 cm^2^ grid.

**Figure 9:**
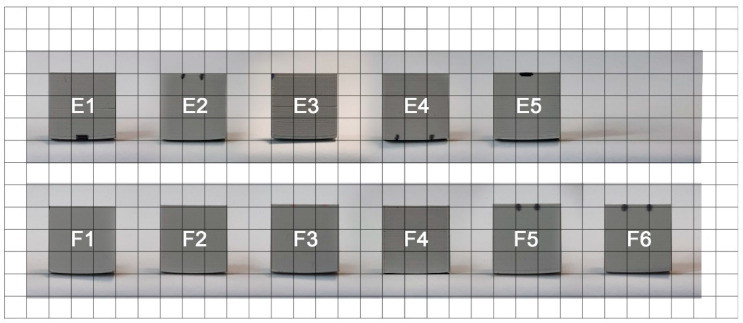
Photos of printed cubes for group E-F, placed on a 1 × 1 cm^2^ grid.

In the first step, the obtained printouts were visually assessed to look for any visible edge deformations or other defects. Three main types of defects were occurring: warping (elevation of the bottom part), cracks (stratifications of filament layers, shown in Figure 6) and other wall deformations. Photos of the printouts are presented in consecutive Figure 7, Figure 8 and Figure 9 and the observations are summarized in Table 3. The D1 color in the photo does not match the filament color because it was base coated with the Army Painter fur brown color primer [26] for the scanning process due to the fact that the EinScan-SP SPECS Desktop 3D Scanner does not register black.

In group A, closer inspection revealed that along with the growth in width the model warped, in the extreme case of A4 forming a banana-shaped defect. Additionally, small cracks start appearing in the wider models, noticeably earlier on the side with larger warping. These findings contradict some recommendations from Markforged et al. [27], where the authors recommend orienting the model with the largest face turned down. As for group B, the implemented changes in height resulted in warping which level from model to model is debatable in these conditions, however, cracks themselves in higher printouts appear in greater numbers and starts on lower levels of the print. In addition, cracks are larger on the side with bigger warping, another representation of the conclusions of Zgyza et al. [21]. No conclusive results could be formulated for group C due to the fact that no trend could be found, as C2 was the most deformed printout. This contradicts observations made by Terekhina et al. [22] and it is not clear whether their recommendation to print in the XZ orientation would be beneficial for our models.

For group D (color materials), the darker the color of the filament, the more cracks developed in the printout, a somewhat similar conclusion to Soares [14], where he experimented in the same way, as we did, with different colors of PLA. For group E, all specimens were nearly identical in terms of warping, but not cracking. Lower nozzle temperature produced more cracks that were finally absent after middle of series. This observation is consistent with the empirical data presented by Peng and Wang [23], as well as Xu [28], with both papers pointing out that nozzle temperature is an important factor for print quality. Inspection of group F did not show any noticeable warping changes. What is interesting is the line on the crossing of the infills. It was more prominent where the bed temperature was lower.

### 3.2. Measurements of the Corner Lift

**Figure 10:**
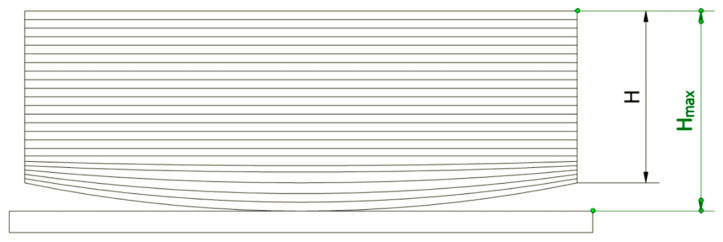
Schematic drawing of vertical edge lift measurements.

**Figure 11:**
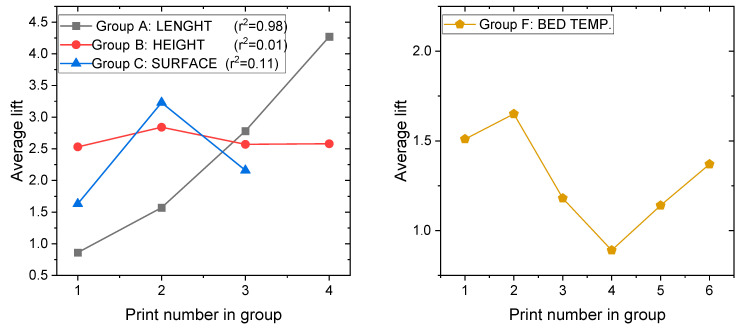
Plots of selected measurements. (**Left**): Groups A–C with correlation coefficients. (**Right**): Group F.

In the next step, the corner lift of each printout was measured along its vertical edges. For this purpose, the maximum height (H_max_) and height along the vertical edge (H) were measured with a caliper for each printout (see Figure 10). The difference between the two values (H_max_-H) was then calculated to obtain the lift value in millimeters. For each cube, the lift of the mean value of the four edges’ lift was calculated together with the standard deviation as a measure of the spread of the results (see Table 4 and Figure 11).

The results of the average lift for group A clearly show that warping increased with the length of the printout. The differences present an increasing dependence; however, determining their exact form, i.e., linear or parabolical, would require producing more intermediate printouts, also with different geometries. The results of the printouts in group B indicate that the vertical dimensions of the printouts do not influence the lift of the bottom edges. This observation seems reasonable, as cracking occurs when the printout layers close to the bed are cooling. Therefore, above some height from the bed new, hot layers from the nozzle could not affect the bottom ones and could only produce cracks. The results are interesting for group C, where the surface of the printout increased. The greatest lift was observed for the middle printout. We think that C1 was too small to produce large cracks and warping, while in C3 the adhesion surface was high enough to minimize the influence of thermal stresses. This is an interesting topic for further investigation; however, it would explain why Terekhina [26] and Markfored [24] presented opposite explanations for the same problem. The results for group A–C are presented in Figure 11 (left), together with corresponding correlation coefficients. One can see that there is a strong positive correlation between length of the printout and warping, while any correlation is observed according to height. The group D results indicate that the composition of the filament material, i.e., the addition of a dye, influences the properties of the printouts. An interesting trend occurred: the smaller lift was observed for darker printouts, while for the brighter ones it got larger. Many factors, such as the required different printing temperatures or the type of dyes, can be involved in shaping the response of the material in this case. Therefore, results are expected to vary between filaments from different companies. This is something that is often neglected in reporting results in the scientific literature, even though the authors often focus on a large number of varying parameters [17,18,27,28]. Groups E and, more expressive, F show a kind of resonance dependence–see Figure 11. The smallest warping happens when the nozzle and bed temperatures are set as proposed by the manufacturer, while both higher and lower temperatures result in bigger lifts. Moreover, in reference [26] researchers empirically concluded that the same bed temperature, as ours, is the best.

### 3.3. Measurements with a 3D Scanner

**Figure 12:**
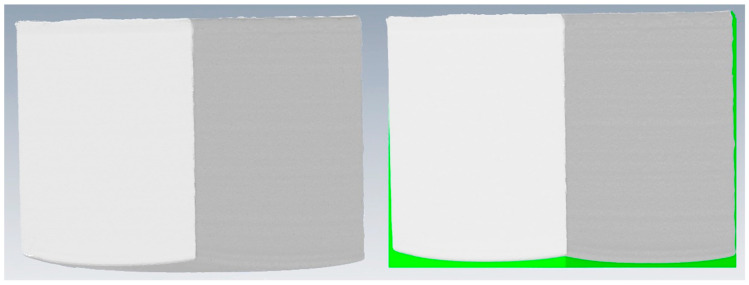
(**Left**): scanned object. (**Right**): Comparison with resized theoretical object. Green parts indicate differences.

After being removed from the printer, all printouts were put into a 3D scanner. This way spatial images of printouts were obtained, from which their volume could be determined. This could not be achieved by manual measurements, e.g., with a caliper, due to the change in dimensions of the samples along the given edges (discussed below) as well as due to the irregular shape of the wrapped base–see Figure 12. All series showed small deviations from the model. Specifically, most printouts had small blow-outs and gouges on their vertical walls, with the exception of E4, which was almost free from such deformations. Series D showed additions of molten polymer on its top, which formed oblique lines. The difference between theoretical (that is, set in a model) and real volume was calculated (Table 5). In general, for small printouts (30 × 30 × 30 mm^3^), their volume was higher than the model predicted, while for the larger ones it was the opposite. Furthermore, the larger the model that was to be printed, the greater the difference between the volume of the computer model and the final printout. Volume losses seem to be understandable and easily explainable by the differences in thermal contraction of the materials used. The volume changes were most interesting for smaller objects. We suppose that it is caused by the accuracy of the printer. The nozzle diameter is 0.4 mm, therefore *every* added filament *line* is this thick. In ideal situation, the printed line will have 0.4 mm, so while printing a surface, the printed edge will exceed the theoretical edge on 0.2 mm. In other words: the printing starts with marking the dimensions of the base and the semi-liquid filament comes out of the nozzle and spreads to the sides so ±0.2 mm this way of thinking is supported by Msallem’s paper [8], where the adherence to the stl file on—for all instances and purposes—the same printer is deviated by a relatively identical value. In other words, in small printouts the printer surplus exceeds the shrinking caused by cooling, while on a larger scale the amount of added filament stays the same, making it less relevant.

### 3.4. Thermal Images

**Figure 13:**
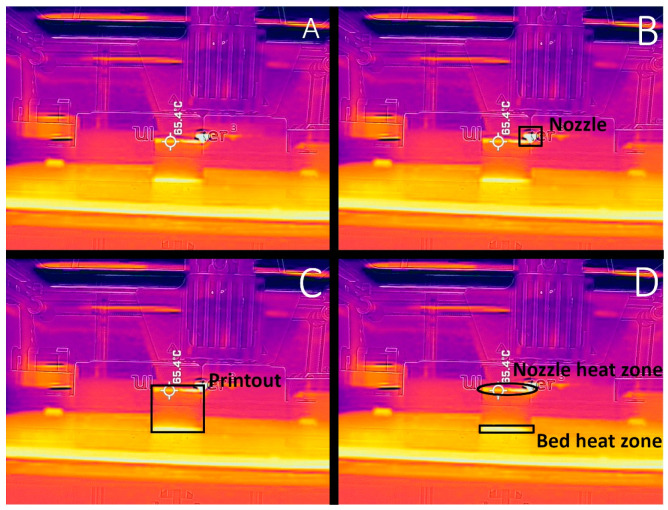
Thermal images obtained during printing: (**A**) printing chamber; (**B**) indicated heat sources—nozzle and bed; (**C**) marked print; (**D**) temperature layout on the printout. Temperature indicated in FLIR software [30].

**Figure 14:**
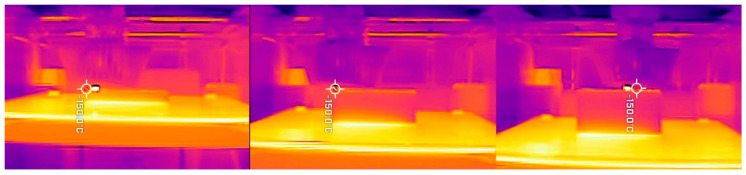
Temperature changes of a printout during printing. Notice that a vertical temperature distribution above some level did not change.

Thermal images have proven to be an invaluable tool in understanding the temperature distribution during printing [12,25,29]. The images of a printing chamber, the model, the bed, and the nozzle give information about temperature differences between all those elements (and the environment of the chamber). Figure 13 shows two distinct heat sources, a small and very hot nozzle (around 250 °C) and a large bed, which occupies the lower half of the (around 90 °C). The space between them is occupied by the printout, which is even cooler than the bed (60–70 °C in the middle). The chamber walls remain relatively unheated at around 20–30 °C. We can clearly see that a few top layers are still hot after exiting from the nozzle (Figure 14), however, they are rapidly cooling off, as the heat zone is not big, as proved by Trejo at al. [12]. This zone is crucial in terms of structural integration of the printout. There are two main heat contraction zones: at the bottom, where the model touches the bed, and at the top. The latter is generated by realizing the hot filament from the nozzle. The thermal model of the printing process was developed by Compton et al. [29]. However, the temperature distribution on the printout is not uniform, nor forms a constant gradient, something Zgryza [25] also observed. The vertical edges are the areas with the lowest temperatures. Also, the crack lines, if present, are noticeably cooler than the neighboring areas. The corners with the largest warping experience the biggest temperature changes confined to the surface directly adjacent to the bed.

**Figure 15:**
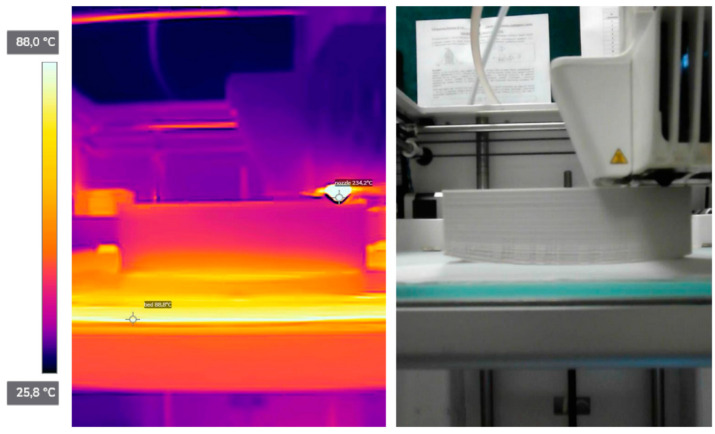
(**Left**): thermal imaging with marked points: nozzle and bed. (**Right**): image taken simultaneously in the visible band.

In the next stage three pictures taken during printing of A3 were analyzed: after 60 min, 120 min and 180 min from the beginning of the printing. Highest temperature registered by a camera was at the nozzle, where it reached 234.2 °C. A 20.8 °C difference between the set printing temperature (255 °C) and resisted is expected because, heatblock (Figure 1), is not visible at the picture. The camera registered the temperature of the glass to be equal to 88.8 °C, while it was set at 90 °C. This difference may be due to the fact that the camera was set to better capture matt surface of the polymer printouts rather than the reflective surface of the glass bed-emissivity was set to 0.8, to capture glass accurately it should be 0.9. For all of the photos, the color scale starts at 25.8 °C and ends at 88.0 °C, which does not take into account the temperature of the nozzle (see Figure 15). This allows for a better visualization of the changes occurring in the printed objects.

**Figure 16:**
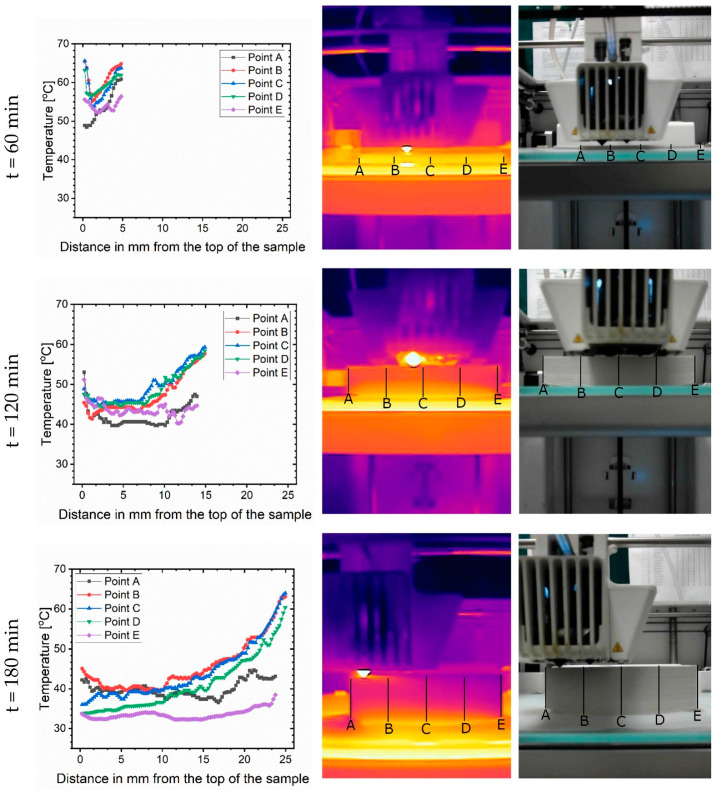
From left to right: temperature profiles, infrared, and visible pictures of the sample at three time points: 60 min, 120 min and 180 min, respectively.

At each picture five temperature profiles of the sample were taken along the vertical lines marked in Figure 16: two at the edges, one in the middle of the sample and two between the above mentioned in both directions. Graphs of these thermal profiles are presented in Figure 16. Temperatures at the edges were lower than in the middle of the sample. This was quite obvious, since these parts could dissipate heat more easily, both due to convection and radiation. Secondly, the temperature of the sample’s base (right end of the graph) was always high due to heating of the bed, but then it dropped sharply. This temperature gradient created stress and peeled off the cooler corners—that is, it led to warping. Third, although the nozzle spitted out hot filament, it cooled down rapidly after meeting a much cooler sample. For t = 120 min and t = 180 min the left edges, i.e., at the top of the sample, were much cooler (mostly below 50 °C). A natural way to reduce the temperature gradient inside the sample is then to minimalize the air flow inside the printing chamber, e.g., by closing it or by printing in a heated chamber. This, however, would require the continuation of the research about the thermal changes in the sample after printing and during cooling.

## 4. Conclusions

**Figure 17:**
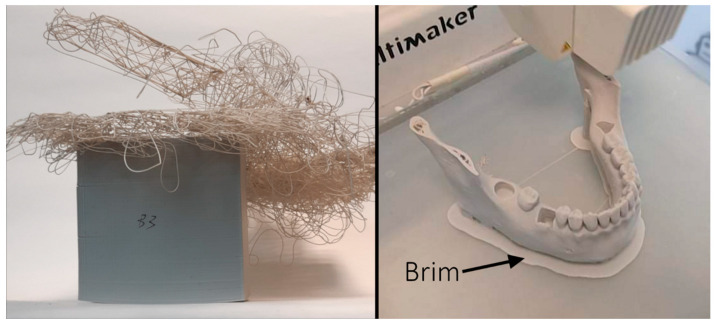
(**Left**): Photo of a sample that broke off the substrate during printing. As a result of warping, the adhesion surface of the sample decreased, which, due to the large moment of force generated by the printing head (high sample-large force arm), resulted in the sample detaching from the bed. This is an example of the negative effects of warping. (**Right**): example of brim, which could reduce warping. Own work.

This paper aimed to assess ABS printed deformation of the objects printed from ABS under various conditions. Various cuboids were printed under different conditions and afterward, the accuracy of the printouts was verified, with special attention on the warping. It was found that the length of the printout increases the risk of deformation, i.e., warping. On the contrary, the height of the object did not significantly affect the warping; however, in higher objects cracks occur due to internal stress. It is noteworthy that the surface of the printout influences the warping nonlinearly. For small objects, it is not significant, with increasing the surface it rises, until some point, when this contact area with hot bad provides stronger adhesion forces, which reduced warping. Additionally, it was shown that the color of the filament might influence the accuracy of the printout as well as the nozzle and bed temperature. For the latest, the smallest deformation was observed while printing at temperatures suggested by the manufacturer. Warping and cracking processes will be always inevitable during 3D printing and will always require careful selection of parameters, so this research might be used as a guide. Summarizing our findings, in order to minimalize deformation of the printed objects, we recommend: firstly, while orienting the printout’s biggest surface area, one should make sure that it adheres to printer’s bed, while also making sure that printout’s height is as short as possible to avoid cracking or detaching from the bed—see Figure 17. Secondly, the nozzle and bed temperature settings should be fine-tuned using the manufacture’s recommended temperature range as the starting point. As shown in this paper, changes such as color will affect overall quality of the print. In addition, while preparing the printing, one could add a bigger flat object, so called “brim” or “raft”, under the initial print and thus enlarge the contact area—see Figure 17.

## Figures and Tables

**Table 1:** Properties of ABS utilized in the study according to the production data sheet [19].

Print Temperature	Normative Diameter	Real Avg. Diameter	Diameter Tolerance	Real Avg. Roundness	Bed Temperature	Density (at 23 °C)
250–265 °C	2850 mm	2848 mm	+/−0.02 mm	0.020 mm	90–110 °C	1.03 g/cm^3^

**Table 2:** Specification of printouts. Group A changes length, group B changes height, group C surface area, group D color, E nozzle temperature, and F bed temperature.

Print Number	Length	Width	Height	Nozzle Temp.	Bed Temp.	Color
A1	30	30	30	255	90	grey
A2	60	30	30	255	90	grey
A3	90	30	30	255	90	grey
A4	120	30	30	255	90	grey
B1	90	30	30	255	90	grey
B2	90	30	45	255	90	grey
B3	90	30	60	255	90	grey
B4	90	30	75	255	90	grey
C1	30	30	30	255	90	grey
C2	60	60	30	255	90	grey
C3	90	90	30	255	90	grey
D1	60	30	30	255	90	black
D2	60	30	30	255	90	grey
D3	60	30	30	255	90	blue
D4	60	30	30	255	90	green
D5	60	30	30	255	90	white
E1	30	30	30	240	90	grey
E2	30	30	30	245	90	grey
E3	30	30	30	250	90	grey
E4	30	30	30	255	90	grey
E5	30	30	30	260	90	grey
F1	30	30	30	255	75	grey
F2	30	30	30	255	80	grey
F3	30	30	30	255	85	grey
F4	30	30	30	255	90	grey
F5	30	30	30	255	95	grey
F6	30	30	30	255	100	grey

**Table 3:** Observations of the deformations of the printout by eye inspection.

Print Number	Observations
A1	Minimal warping on the edges, only visible after close inspection
A2	Visible warping on the left side of the model and few uplifted lines of filament on the back side of the top
A3	Clear warping of all bottom corners, few uplifted lines of filament on the back and front side of the top
A4	visible warping higher on the front
B1	Clear warping of all bottom corners, few uplifted lines of filament on the back and front side of the top side
B2	Small warping on all corners, a little bit higher on the left, 2–3 small cracks on all vertical edges
B3	Higher warping on the left side, 3–5 small cracks on all vertical edges with one big on each edge, and few uplifted lines of filament on the front side of the top
B4	Small warping on all corners, biggest on the front left corner. Several cracks on all vertical edges, the biggest one occupying the entire right wall and around 1/6 of the adjacent walls
C1	Minimal warping in the front, small in the back, no visible cracking
C2	Small warping on all corners, with a single small crack on all vertical edges
C3	small warping on all corners
D1	Barely noticeable warping on all bottom corners, except rear right, where there are small, few uplifted patches of filament in the edges of the top side.
D2	Visible warping on the left side of the model and few uplifted lines of filament on the back side of the top
D3	Small warping on all bottom corners, except the rear right where it is bigger, few uplifted patches of filament in the edges of the top
D4	Warping on all bottom corners, larger in the front, and few uplifted patches of filament in the edges of the top side
D5	Small warping on all bottom corners, except rear left, where it is bigger, few uplifted patches of filament in the edges of the top
E1	Minimal warping on the edges, only visible after close inspection, crack on the front right vertical edge
E2	Minimal warping in the front corners, small warping on the back ones
E3	Minimal warping in all corners except rear left where is small
E4	Barely noticeable warping in the bottom corners, slight under extrusion of the rear right corner
E5	Barely noticeable warping on the bottom corners, right bottom edge deformed
F1	Barely noticeable warping in the bottom corners, except in the rear right corner with small warping
F2	Barely noticeable warping in the bottom corners
F3	Barely noticeable warping in the bottom corners
F4	Barely noticeable warping in the bottom corners
F5	Barely noticeable warping in the bottom corners, except for the rear right with small warping
F6	Barely noticeable warping on the bottom corners

**Table 4:** Results of the measurements of average vertical lift for each printout in mm.

	Results					
Print number	A1	A2	A3	A4		
Average lift (SD)	0.86 (24)	1.57 (52)	2.78 (31)	4.27 (46)		
Print number	B1	B2	B3	B4		
Average lift (SD)	2.53 (51)	2.84 (60)	2.57 (95)	2.58 (11)		
Print number	C1	C2	C3			
Average lift (SD)	1.63 (15)	3.23 (51)	2.16 (41)			
Print number	D1	D2	D3	D4	D5	
Average lift (SD)	1.97 (59)	1.66 (54)	2.00 (77)	2.28 (79)	2.60 (90)	
Print number	E1	E2	E3	E4	E5	
Average lift (SD)	1.55 (54)	1.36 (37)	1.80 (22)	1.22 (41)	1.26 (45)	
Print number	F1	F2	F3	F4	F5	F6
Average lift (SD)	1.51 (36)	1.65 (45)	1.18 (35)	0.89 (31)	1.14 (45)	1.37 (46)

**Table 5:** Results of the differences between the theoretical and real volume of the printouts.

	Results					
Print number	A1	A2	A3	A4		
V_measured_/V_theoretical_	1.03	0.99	0.98	0.93		
Print number	B1	B2	B3	B4		
V_measured_/V_theoretical_	0.98	0.97	0.92	0.91		
Print number	C1	C2	C3			
V_measured_/V_theoretical_	1.01	0.98	0.98			
Print number	D1	D2	D3	D4	D5	
V_measured_/V_theoretical_	1.04	1.06	0.99	1.04	0.95	
Print number	E1	E2	E3	E4	E5	
V_measured_/V_theoretical_	0.98	1.04	0.94	0.98	0.99	
Print number	F1	F2	F3	F4	F5	F6
V_measured_/V_theoretical_	0.97	0.97	0.97	0.99	0.99	1.03

## Data Availability

The data presented in this study are contained within the article. Any additional information and data are available on request from the authors.
